# Construction of homogeneous antibody–drug conjugates using site-selective protein chemistry

**DOI:** 10.1039/c6sc00170j

**Published:** 2016-02-12

**Authors:** Padma Akkapeddi, Saara-Anne Azizi, Allyson M. Freedy, Pedro M. S. D. Cal, Pedro M. P. Gois, Gonçalo J. L. Bernardes

**Affiliations:** a Instituto de Medicina Molecular , Faculdade de Medicina , Universidade de Lisboa , Avenida Professor Egas Moniz , 1649-028 Lisboa , Portugal . Email: gbernardes@medicina.ulisboa.pt; b Department of Chemistry , University of Cambridge , Lensfield Road , CB2 1EW Cambridge , UK . Email: gb453@cam.ac.uk; c Research Institute for Medicines (iMed.ULisboa) , Faculty of Pharmacy , Universidade de Lisboa , Lisbon , Portugal

## Abstract

Systemic chemotherapy, the current standard of care for the treatment of cancer, is rarely curative and is often accompanied by debilitating side effects. Targeted drug delivery stands as an alternative to chemotherapy, with the potential to improve upon its low efficacy and systemic toxicity. Among targeted therapeutic options, antibody–drug conjugates (ADCs) have emerged as the most promising. These conjugates represent a new class of biopharmaceuticals that selectively deliver potent cytotoxic drugs to cancer cells, sparing healthy tissue throughout the body. Despite this promise, early heterogenous ADCs suffered from stability, pharmacokinetic, and efficacy issues that hindered clinical development. Recent advances in antibody engineering, linkers for drug-release, and chemical site-selective antibody conjugation have led to the creation of homogenous ADCs that have proven to be more efficacious than their heterogeneous predecessors both *in vitro* and *in vivo*. In this minireview, we focus on and discuss recent advances in chemical site-selective modification strategies for the conjugation of drugs to antibodies and the resulting potential for the development of a new generation of homogenous ADCs.

## Abstract 2

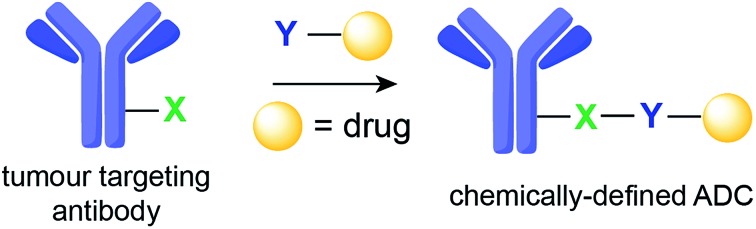
The use of site-selective chemical drug-conjugation strategies enables the construction of antibody–drug conjugates (ADCs) with superior therapeutic efficacy.”

## Introduction

**Fig. 1:**
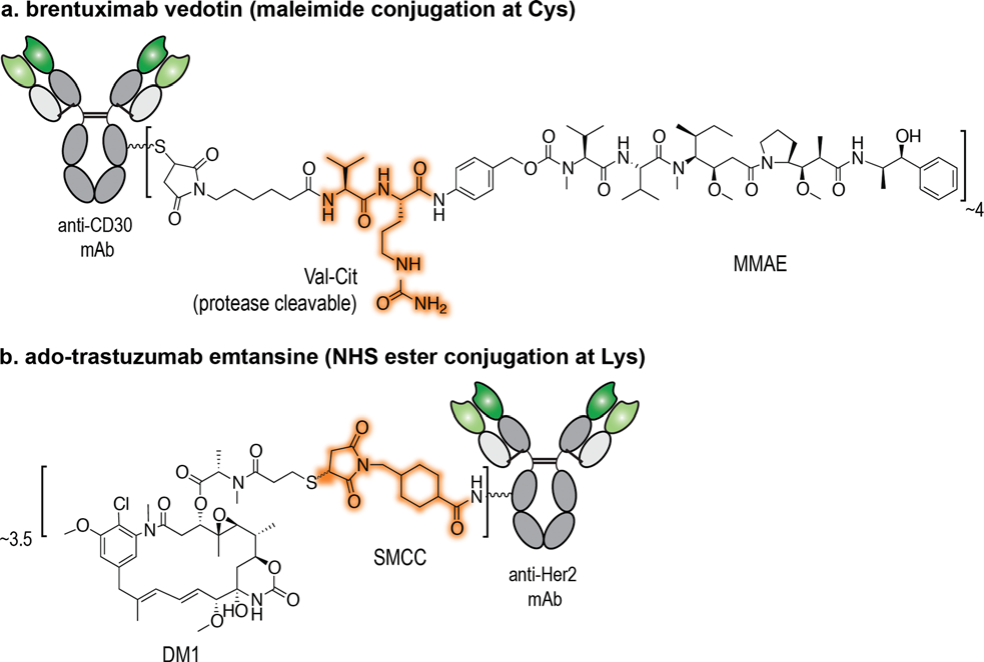
Structures of currently FDA approved ADCs. (a) Brentuximab vedotin (Adcetris®; Seattle Genetics/Millennium Pharmaceuticals);^4^ and (b) ado-trastuzumab emtansine (Kadcyla® – T-DM1; Roche/Genentech).^5^ NHS ester: *N*-hydroxysuccinimide ester; Val-Cit linker: valine-citrulline linker; SMCC: succinimidyl-4-[*N*-maleimidomethyl]cyclohexane-1-carboxylate; DM1: thiol-containing maytansinoid. MMAE: monomethyl auristatin E.

Antibody–drug conjugates (ADCs) represent one of the most promising strategies for the selective targeting and delivery of cytotoxic drugs to malignant tumour cells.^1^–^3^ Typically comprised of a tumour-recognizing monoclonal antibody (mAb) derivative linked to a highly potent cytotoxic drug, ADCs combine the targeting ability of the mAb with the lethality of a cytotoxic drug (Fig. 1). This results in tissue selectivity and improved efficacy of treatment, provided that the chemical linker maintains the integrity of the conjugate in circulation. Despite early recognition of this potential, the initial development of ADCs progressed slowly, limited by the number of tumour-specific targets, the stability of conjugates, and the efficacy and pharmacokinetics of relatively large intact mAbs (MW ∼ 150 kDa).^2^ Currently, Seattle Genetic's Adcetris® for treatment of relapsed Hodgkin Lymphoma (Fig. 1a)^4^ and Roche's Kadycla® for HER2 positive metastatic breast cancer (Fig. 1b)^5^ are the only Food and Drug Administration (FDA) approved ADCs on the market.

However, in recent years, targeting and technical discoveries have dramatically improved the therapeutic utility of ADCs and led pharmaceutical companies to embrace ADC technology; today, more than 30 ADCs have progressed to clinical trials.^6^ The increasing number of clinically relevant antigen targets has expanded the potential range of ADC activity,^7^ while advances in antibody engineering address some of the issues with mAbs. Smaller recombinant antibody formats, such as single chain variable fragments (scFv) or nanobodies, accumulate rapidly and in high concentrations in tumours, improving binding and penetration.^8^ Moreover, chemical linker technology has improved as well, enabling the release and activity of the cytotoxic drug only upon target engagement.^2^ Finally, many of the initial problems facing ADCs, including potency, stability, and toxicity, were linked to heterogeneity – that is, the variances in the location and number of cytotoxic molecules linked to the antibody. The development of efficient, site-selective chemical conjugation strategies has allowed for the emergence of homogenous ADCs with superior therapeutic properties.^9^

In this minireview, we focus on a key component of ADC design: antibody–drug conjugation technology. We describe recent examples of homogenous ADCs built using chemical site-selective antibody conjugation methodologies – *i.e.*, chemical transformations that preferentially modify one amino acid residue over the others (*e.g.*, cysteine (Cys) over lysine (Lys)) – and discuss how such methodologies have been and may be used to provide ADCs with improved safety, selectivity, and efficacy profiles.^10^ In addition, we also highlight methods that have thus far only been used on proteins, but hold potential for the construction of homogenous ADCs. The methods covered in this minireview are chemical ligation reactions between the side chain of a natural, non-canonical amino acid or at glycosylation sites and a suitable drug derivative. Methods based on enzymatic ligation, such as the use of bacterial transglutaminases,^11^ have been covered elsewhere.^12^

## Drug conjugation technology

The ligation of a linker bearing a cytotoxic drug to a mAb is an essential step in the construction of ADCs. Early strategies to establish this conjugation involved the direct functionalization of abundant solvent-accessible Lys residues using *N*-hydroxysuccinimide (NHS) ester derivatives. This method of conjugation often resulted in the generation of heterogeneous conjugates with varying pharmacokinetic and therapeutic properties.^13^–^15^ For these reasons, the construction of chemically-defined conjugates has emerged as a key goal of ADC design, leading to the development of methodologies that enable the site-selective chemical modification of mAb. In one example, Junutula and co-workers demonstrated that an ADC labelled non-specifically at Lys residues resulted in a lower efficacy when compared to the same mAb labelled site-selectively at an engineered Cys (as indicated by a greater reduction in tumour volume).^9^

Cys is now the primary residue target to achieve site-selective conjugation of drugs to mAbs, due to low Cys abundance and the enhanced nucleophilicity of its sulfhydryl side chain.^16^ Such methods often rely on the engineering of additional free Cys by site-directed mutagenesis and/or reduction of existing disulfide bridges and their further use for conjugation.^17^ Nevertheless, alternative approaches for the construction of homogeneous conjugates are now being pursued, namely the chemical modification of non-canonical amino acids genetically introduced into the mAb's structure.^12^ Finally, full-length IgG mAbs display a glycan at the conserved asparagine (Asn) 297 residue in the CH2 domain. A number of glycoengineering-based strategies have also been pursued for the attachment of drugs to the antibody through this motif.^12^ No matter the method of chemical modification, the conjugation of the linker-drug moiety to the mAb should proceed rapidly under mild conditions (neutral pH, buffered solution, room temperature) and lead to the formation of a homogenous ADC while retaining the structural integrity and antigen binding capacity of the mAb.

In the sections below, we review strategies that have been used to conjugate drugs to mAbs and highlight technologies that may find utility in the generation of homogenous and plasma stable ADCs in the future. For further reading on general site-selective chemical methods for the modification of proteins, we direct the readers to a number of recent reviews in the field.^18^–^20^

## Conjugation strategies at natural amino acids

### Lysine modifications

Several modifications of the ε-amine of Lys residues of proteins have been reported *via* reactions with isothiocyanates (NCS), *N*-hydroxysuccinimidyl (NHS) esters, anhydrides, fluorophenyl esters, aldehydes (whose products are stabilized by functional groups present in the molecule or with a subsequent reduction), and activated lactams.^21^ Two of the ADCs that were approved by the FDA, gemtuzumab ozogamicin and ado-trastuzumab emtansine (Fig. 1b), were assembled using Lys conjugation protocols. NHS ester reagents were originally the most common choice for building ADCs through Lys conjugation. NHS esters have been used to introduce bioorthogonal functionalities, such as azides and hydrazones, for subsequent modification through Staudinger ligation and hydrazone exchange, respectively.^22^–^24^ NHS esters can also be used to modify maleimide-crosslinkers. These crosslinkers can then be used to conjugate antibodies to different molecules bearing a nucleophilic sulfhydryl functional group, such as drugs, quantum dots, and even DNA barcodes.^25^–^29^

Though widespread, the application of Lys/activated ester conjugation protocols is accompanied by a number of limitations. Not only can this approach result in modification of the *N*-terminus, it can generate two-fold heterogeneous products. In the case of huN901-DM1 conjugate, the mAb contains 86 Lys residues. Upon reaction with the activated NHS ester, the drug found to be distributed over 47% of the 86 Lys residues present on the mAb. The mixture contained over 4.5 million unique molecules with a drug-to-antibody ratio (DAR) ranging from 0 to 6, while regioisomers with the same DAR were found as well.^15^ In fact, both gemtuzumab ozogamicin and ado-trastuzumab emtansine conjugates are present in heterogeneous mixtures containing 0 to 8 drug moieties per antibody, with an average DAR of 3 to 3.5.^13^,^30^ Moreover, these succinimidyl ester cross-linking reagents can undergo cross-reactivity with tyrosine (Tyr), though the reactivity of Lys over Tyr can be controlled by solvent accessibility, abundance of the residues, and the reaction conditions pH. At acidic pH 6, Tyr is more reactive than Lys, while at alkaline pH 8.4, Lys modification predominates.^31^

Other more selective methods of Lys based modification have been explored with success. For instance, a DOTAGA-trastuzumab conjugate was generated *via* an anhydride conjugation between a humanized mAb (trastuzumab) and a chelating agent (DOTA) possessing a cyclic anhydride suitable for conjugation with the primary amine side chain of Lys residues.^32^ An activated β-lactam bearing an analogue of an anti-viral drug was used to modify mAb 38C2 at a Lys residue as well.^33^ Furthermore, fluorophenyl ester drug analogues were used to modify an anti-CD90 antibody (5 × 10^10^),^22^ while isothiocyanates were used to introduce a radioactive label in anti-CD45 mAb through conjugation to the amine side chains of Lys residues.^34^

Despite some therapeutic achievements, the conventional Lys-based conjugation of drugs to antibodies, in particular the use of NHS esters, generates heterogeneous ADCs with potentially different pharmacokinetics and therapeutic efficiency. These challenges have inspired the development of novel and robust methodologies for antibody conjugation resulting in homogenous ADCs, some of which are discussed in the following sections.

### Cysteine modifications

**Fig. 2:**
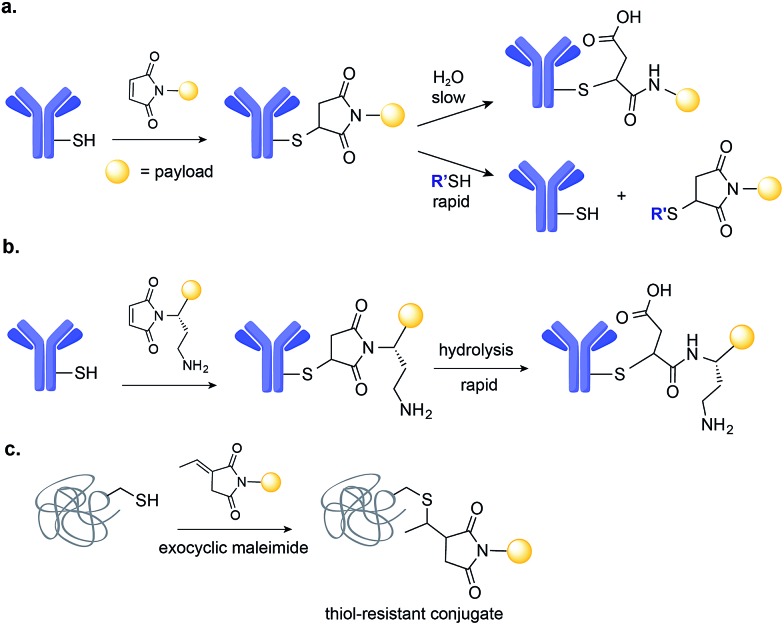
Maleimide-based drug conjugation. (a) Maleimide conjugation leads to a thiosuccinimide adduct that can undergo either rapid retro Michael-addition reaction or slow hydrolysis. (b) Placement of a basic amino group adjacent to the maleimide promotes intramolecular thiosuccinimide ring hydrolysis. (c) The use of exocyclic maleimides derivatives as opposed to conventional endocyclic ones results in fully thiol-exchange resistant product.

The conjugation of maleimides to Cys residues on mAbs is currently the method of choice for the assembly of ADCs (Fig. 2a). Maleimide-bearing linkers are synthetically accessible and demonstrate selectivity for the sulfhydryl side chain of Cys, as well as rapid ligation kinetics in aqueous conditions.^35^ In fact, the efficiency and selectivity of this methodology led it to find utility beyond the conjugation of drugs to mAbs; it has been used for the introduction of photoactivatable functionalities, radiohalogen chelator groups, and nanoparticles.^36^–^38^ Antibody fragments and affibodies, antibody mimetics, have also been modified using this technology.^39^–^41^

Initial methodologies for the modification of IgG antibodies relied on the reduction of the interchain disulfides, followed by careful re-oxidation to leave a single pair of Cys free for further conjugation with maleimide reagents.^42^ This process is extremely difficult to control, often leading to the formation of mixtures and loss of integrity of the antibody structure, therefore reducing antigen binding affinity. This problem can be addressed through the addition of Cys residue(s) through site-directed mutagenesis, which may then be modified without disrupting the native interchain disulfides and thus antibody structure and binding. In one example, Cys residues were placed at defined positions to control reactivity towards maleimides and decrease conjugate heterogeneity. These rationally designed, site-selectively modified antibodies, designated THIOMABs, have shown improved *in vivo* efficacy and safety.^9^ While the first THIOMABs were used to generate ADCs with a DAR of 2, the same strategy has now been used to build ADCs with a DAR of 4. These ADCs with higher drug loading showed improved therapeutic activity when compared to the same ADC with a DAR of 2.^43^

Applications of maleimide conjugation technology for construction of ADCs have been widely explored. Most notably, brentuximab vedotin, an FDA-approved ADC, was synthesized *via* Cys conjugation (Fig. 1a). This ADC couples a chimeric anti-CD30 mAb (cAC10) and the anti-mitotic agent MMAE through a protease-sensitive valine-citrulline linker.^44^ Of course, maleimide-based conjugation faces its own challenges. Maleimide reagents may cross react with other functional groups in a protein, mainly the ε-amino group of Lys – though under typical conjugation conditions (0.001 M maleimide, aqueous buffered solution at pH 7), the reaction with the Cys sulfhydryl is usually 1000 times faster than with the amino side chain of Lys.^45^ More problematically, the resulting thioether succinimide motif ultimately degrades *in vivo* by rapid reaction with reactive thiols in plasma through retro-Michael addition reactions (Fig. 2a). This degradation results in the systemic release of the cytotoxic drug and consequently, lower efficacy and increased side-toxicity.^17^ However, the slower alternative reaction, hydrolysis of the thiosuccinimide ring, forms an elimination-resistant derivative. Promoting thioether hydrolysis provides a strategy for stabilization of maleimide-conjugated ADCs and precludes early, non-selective drug release.^46^,^47^ In one instance, this was achieved by engineering sites with positively charged amino acids adjacent to the thioether, thereby favouring the formation of the more stable hydrolysis adduct instead of the retro-Michael addition product.^48^ More recently, rapid thioether succinimide hydrolysis was achieved by introducing an adjacent basic amino group that promoted intramolecular hydrolysis of the thiosuccinimide ring and thus avoided deconjugation of the cytotoxic drug (Fig. 2b).^49^ The resulting ADCs have proved to be more stable in plasma and more efficacious *in vivo*, while also evincing fewer side effects than traditional maleimides.^49^ As an alternative to modified maleimides that undergo rapid hydrolysis, exocyclic olefinic maleimide reagents have been reported to allow for rapid and selective Cys modification while forming a linkage that resists thiol-exchange-mediated cleavage.^50^ However, this technology has only been demonstrated on single Cys-containing proteins (Fig. 2c).

**Fig. 3:**
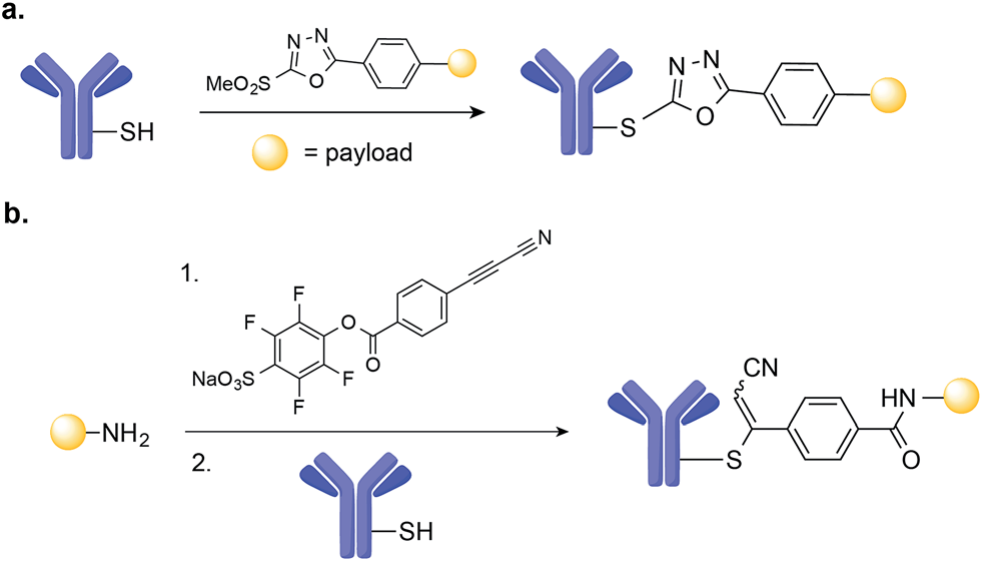
New methods for the chemical site-selective modification of engineered Cys on the surface of mAb based on: (a) Julia–Kocieński-like reagents, such as methylsulfonylphenyloxadiazole. (b) Amine-to-thiol coupling using a heterobifunctional reagent, sodium 4-((4-(cyanoethynyl)benzoyl)oxy)-2,3,5,6-tetrafluorobenzenesulfonate.

A number of novel methods for the selective modification of Cys residues have been reported in recent years.^17^ The reaction of Cys with Julia–Kocieński-like reagents such as phenyloxadiazole sulfone derivatives (Fig. 3a),^51^ and with 3-arylpropiolonitriles (Fig. 3b)^52^ havs been used to construct homogenous ADCs with improved stability in human plasma, as compared to typical ADCs prepared *via* maleimide conjugation.^53^,^54^

Other Cys-based modification strategies involve the reaction of the Cys thiol group with known Michael acceptors, such as vinyl sulfones,^16^ allenamides,^55^ and α-halocarbonyl compounds (including perfluoroaromatic molecules^56^ and mono-bromomaleimides).^57^ In addition, the conversion of Cys into dehydroalanine (Dha), followed by thiol Michael-addition, provides access to a thioether-linked conjugate that is stable even in the presence 10 mM glutathione (GSH).^58^,^59^ Thioether conjugates may also be accessed through free-radical thiol-ene coupling.^60^ However, many of these methods have only been used for the modification of Cys on the surface of proteins; their utility for the construction of ADCs and suitability for *in vivo* applications is yet to be demonstrated.

Engineering the addition of Cys residues on the surface of an antibody, in a location that does not negatively impact binding affinity, is a powerful strategy for the preparation of homogenous ADCs when coupled with Cys-selective conjugation methods. We anticipate that in the future, a number of these methods will be used to construct safer, more efficacious ADCs with optimal plasma stability.

### Disulfide bridge modifications

**Fig. 4:**
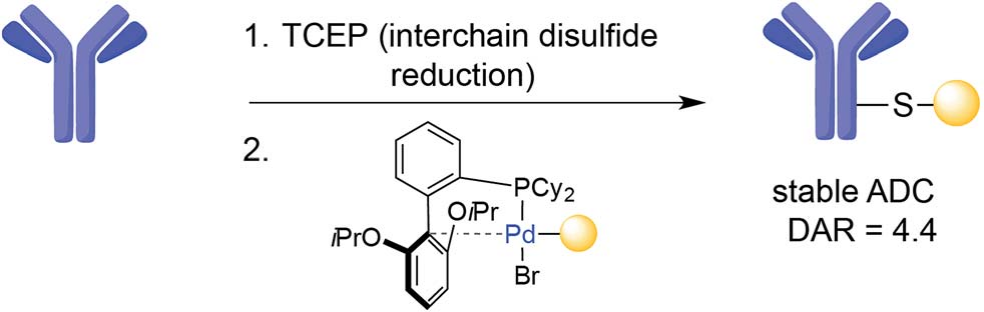
Example of Cys arylation using an organometallic palladium reagent on trastuzumab.

The interchain disulfides of full-length IgGs offer an opportunity for the selective addition of drugs to antibodies. One strategy is disulfide reduction and subsequent introduction of modifications at the reduced Cys residues *via* alkylation.^42^ The reduction–alkylation of mAbs is convenient because it does not require antibody engineering, but controlled reduction of a particular disulfide while maintaining other interchain disulfides is a difficult task. Heterogeneous conjugates and loss of structural integrity and antigen-binding capacity of the mAb are often a result of disulfide-based conjugation strategies. However, Senter and co-workers recently reported an ADC with a DAR of 8 that was assembled by Cys-alkylation of the eight Cys residues following global reduction of interchain disulfides using tris-carboxyethylphosphine (TCEP). In this case, the resulting ADC was shown to be homogeneous and possess both stability in plasma and potent anti-tumour effects in a xenograft mouse model of cancer.^61^ In addition, a transition-metal-based reaction has also recently been introduced for the modification of Cys residues resulting from interchain disulfide reduction on trastuzumab. This method uses palladium(ii) complexes to form stable aryl bioconjugates under mild conditions that maintained the binding capacity of the native antibody (Fig. 4).^62^

**Fig. 5:**
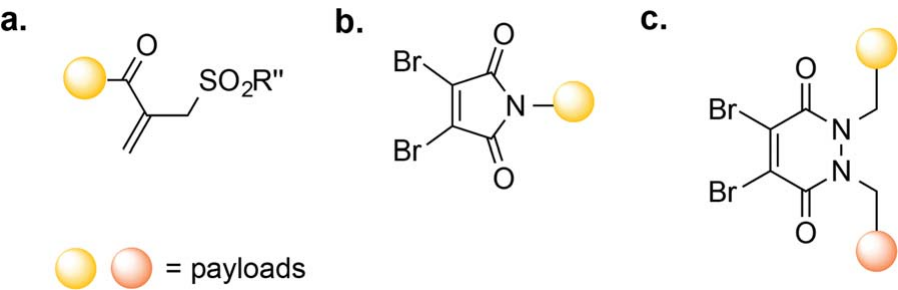
Disulfide re-bridging methods for antibody-conjugation based on (a) vinylsulfone; (b) dibromomaleimide and (c) dibromopyridiazinedione reagents.

Disulfide rebridging, the insertion of small organic molecules into the disulfide bond that render it stable to reduction, presents an alternative to reduction–alkylation. In one example, disulfide rebridging was achieved using a cross-functionalised sulfone PEG reagent (Fig. 5a). Michael-addition of one free thiol to the sulfone forms sulfinic acid and a conjugated double bond, enabling attack of the second thiol and yielding a three-carbon bridge between the two sulfur atoms. This strategy was used for the site-selective PEGylation of human interferon α-2b, as well as for the modification of a fragment of an anti-CD4 antibody.^63^ Similarly, MMAE with a sulfone reagent was prepared and conjugated to trastuzumab, yielding a homogenous ADC with a DAR of 4. This ADC was shown to be highly stable in plasma, to retain antigen affinity, and to promote a strong anti-tumour effect in mice.^64^

Another class of reagents that have been shown to successfully re-bridge reduced disulfides are dibromomaleimides.^57^ In one application, dibromomaleimides equipped with an electron paramagnetic resonance (EPR) spin-label were used to modify an antibody fragment, enabling antigen detection by continuous-wave EPR (cw-EPR).^65^ Dibromomaleimides have also been used to produce homogenous ADCs through the re-bridging of reduced interchain disulfide bonds (Fig. 5b).^66^ One such ADC consisted of the mAb trastuzumab linked to the cytotoxic doxorubicin (DOX); this ADC possessed a defined DAR and successfully bound HER-2.^66^ Reagents termed next generation maleimides (NGM) have also been used to construct a stable, potent, and selective trastuzumab-MMAE ADC *in vitro*.^67^ More recently, Chudasama, Caddick, and co-workers reported the use of dibromopyridazinediones (Fig. 5c) as an alternative to dibromomaleimides, allowing the introduction of two different payloads on the same antibody.^68^

While the above presented strategies allow for the production of homogenous, functional ADCs, the efficacy and safety of the resulting conjugates have not been tested *in vivo*, and thus, their full potential is unknown.

### *N*- or *C*-terminus modifications

**Fig. 6:**
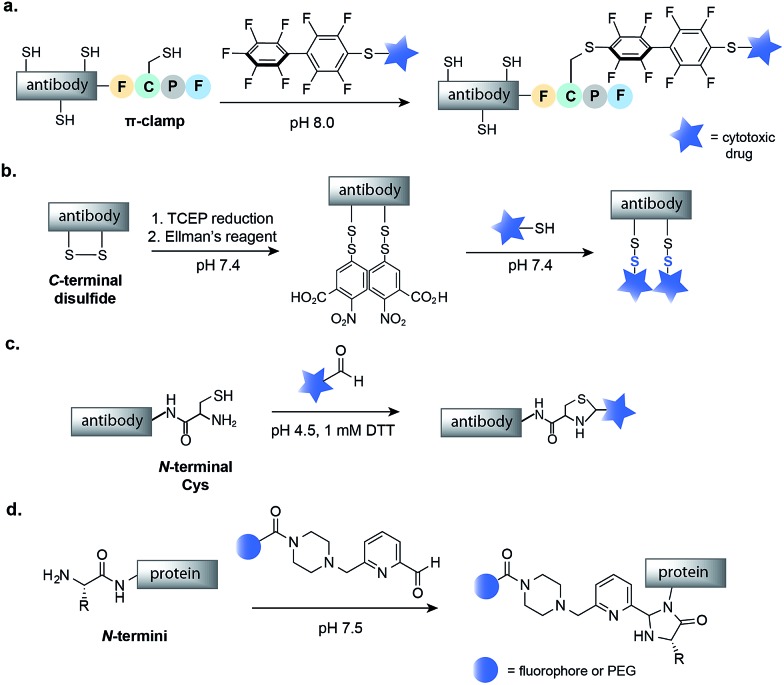
Methods for the chemical modification of IgGs, antibody fragments and proteins at the *N*- or *C*-termini. (a) π-Clamp-mediated antibody conjugation using perfluoroaromatic reagents. (b) Mixed disulfide formation of *C*-terminal Cys. (c) Thiazolidine modification of *N*-terminal Cys with aldehyde containing drugs. (d) *N*-Terminal imidazolidinone formation using 2-pyridinecarboxyaldehyde reagents. TCEP–Tris(2-carboxyethyl)phosphine; DTT–dithiothreitol.

The *N*- and *C*-termini of engineered IgGs and smaller antibody fragments offer another possible site for drug conjugation. Such terminal residues are particularly attractive because they are distant from binding domains, and therefore, modifications should not interfere with antibody binding. In order to allow for site-selective conjugation, Cys residues can be engineered at the *C*- or *N*-terminus of IgGs or smaller antibody fragments such as diabodies (Dbs) or small immune proteins (SIPs). In one recent example, Pentelute and co-workers have reported a four-amino-acid sequence (Phe-Cys-Pro-Phe), which they called the ‘π-clamp’, that modulates the reactivity of the sulfhydryl side chain of cysteine for site-selective conjugation with perfluoroaromatic reagents.^69^ This strategy was demonstrated on trastuzumab, which was engineered with the Phe-Cys-Pro-Phe amino acid sequence at the *C*-terminus (Fig. 6a). Site-selective conjugation with a perfluoroaromatic MMAE derivative resulted in a homogenous conjugate that retained the binding activity of the native antibody and enabled selective killing of HER2-positive breast cancer cells.

The selective modification of antibodies has also been achieved at engineered disulfides at the *C*- or *N*-termini of antibodies. Neri and co-workers have developed a protocol for mixed disulfide drug conjugation to vascular-targeting antibodies (full length IgGs, SIPs and Dbs) with engineered disulfides at the *C*-terminus.^70^,^71^ Following mild reduction of the disulfide with TCEP, activation of the Cys residues with Ellman's reagent and attack of a thiol-containing drug, such as a thiol derivative of cemadotin or DM1, rapidly form a mixed disulfide linkage (Fig. 6b). The resulting non-internalizing ADCs were shown to be stable in human plasma and to cure cancer in immunocompetent mice.^72^ Interestingly, it has been shown that the stability, and consequently the efficacy, of such conjugates is dependent on the distance between the modified Cys residue and the globular fold of the antibody.^73^ The same research group also demonstrated that a diabody fragment with an engineered *N*-terminal Cys residue can selectively react with the aldehyde moiety of a drug to yield a thiazolidine-linked conjugate (Fig. 6c).^74^ Such thiazolidine-linked conjugates have been shown to slowly release the cytotoxic under slightly acidic conditions, though *in vivo* testing has not yet been reported.

Finally, there has been much work on modification of proteins at the *N*-termini that has not yet been applied to the assembly of ADCs.^18^ For example, Francis and co-workers have recently reported the *N*-terminal modification of proteins using 2-pyridinecarboxyaldehydes (Fig. 6d), a methodology that maintains the structural integrity of proteins and could be used to construct a safe and efficacious ADC.^75^ Thus, future work could use such methodologies to build homogenous ADCs.

## Conjugation strategies at non-canonical amino acids

During the last decade, progress in residue-specific genetic encoding of non-canonical amino acids into proteins has expanded the methods available for protein site-selective chemical modification.^76^ Of particular significance was the development of new orthogonal amino-acyl-tRNA synthetase/tRNA pairs, which enable the incorporation of a large number of non-canonical amino acids bearing ketone, aldehyde, azide, alkyne, alkene, tetrazine, aryl halide, or boronate functional groups into *E. coli*, yeast, and even mammalian cells.^77^,^78^ In fact, the incorporation of non-canonical amino acids, in particular ketones and aldehydes, into antibodies has already been used to construct homogenous ADCs with potent anti-tumour efficacy.^79^,^80^

**Fig. 7:**
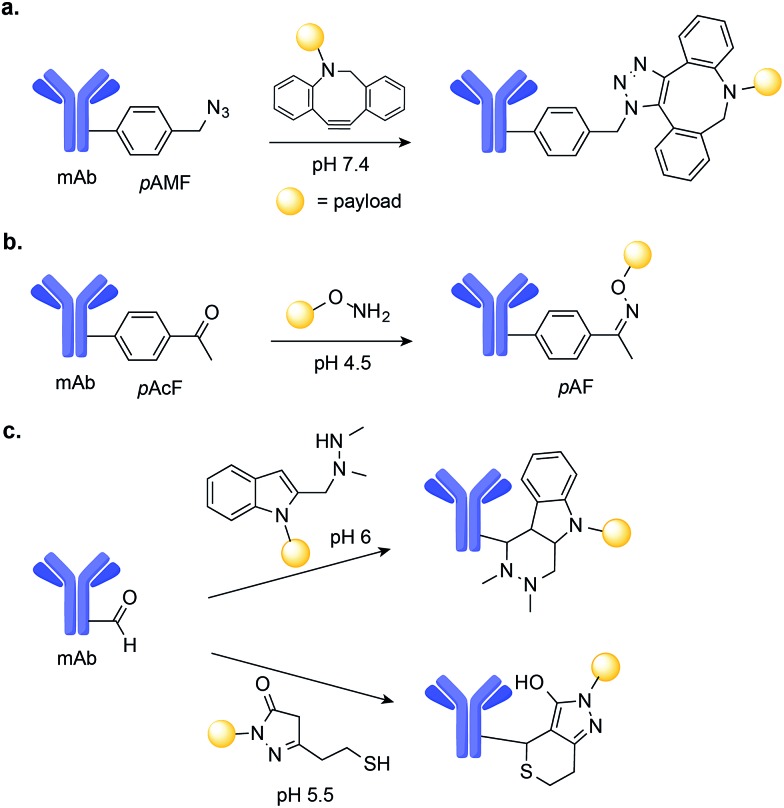
Chemical site-selective modification of non-canonical amino acids on mAbs. (a) Genetic encoding of *p*-azidomethyl-l-phenylalanine (*p*AMF) into a Her2-binding IgG trastuzumab allows for efficient drug conjugation through SPAAC. (b) Chemical site-selective oxime ligation at non-canonical amino *p*-acetyl-l-phenylalanine (*p*AcF) tagged mAb. (c) Aldehyde-tagged mAbs may be selectively modified either by (i) Hydrazino-*iso*-Pictet-Spengler (HIPS) or (ii) trapped-Knoevenagel ligation.

In one example, Sato and co-workers used a cell-free expression system to genetically encode the azido-containing *p*-azidomethyl-l-phenylalanine (*p*AMF) into a HER2-binding IgG trastuzumab.^81^ The azido group was then selectively modified with dibenzocyclooctylpolyethylene glycol monomethylauristatin F (DBCO-PEG-MMAF) using strain-promoted azide-alkyne cycloaddition (SPAAC) copper-free click chemistry (Fig. 7a). The resulting ADC proved to be highly potent in cell cytotoxicity assays.^81^

Schultz and co-workers used an orthogonal amino-acyl-tRNA synthetase/tRNA pair to encode the ketone-containing *p*-acetyl-l-phenylalanine (*p*AcF) into trastuzumab and an anti-CXCR4 antibody.^82^,^83^ This ketone tag can selectively react with alkoxy amine-functionalized auristatin under acidic conditions (pH 4.5) to form an oxime linkage and produce homogenous ADCs (Fig. 7b). These ADCs were shown to be stable in plasma and when administered in xenograft models of cancer, to lead to cures.^80^,^83^ Additionally, when compared with ADCs comprised of the same antibody–drug pair instead linked through Cys–maleimide conjugation, the ADCs generated through oxime-ligation at *p*AcF showed enhanced *in vivo* safety and efficacy, demonstrating the value of this approach.^80^

Aldehydes are another unnatural amino acid handle that can be introduced in mAbs to achieve site-selective drug-conjugation. Formylglycine-generating enzyme (FGE) can be used to residue-specific incorporation of a formylglycine (fGly) residue into a protein scaffold through the selective oxidation of a thiol embedded in the FGE recognition sequence.^84^,^85^ Using this chemoenzymatic approach to introduce fGly, Rabuka and co-workers have developed the Hydrazino-*iso*-Pictet-Spengler (HIPS)^86^ conjugation method, which uses an alkylhydrazine nucleophile to attack the aldehyde instead of the aminooxy nucleophile used for conventional Pictet–Spengler ligation.^87^ The HIPS ligation proceeds at near neutral pH and results in the formation of a stable C–C linkage (Fig. 7c).^88^ When applied to the construction of ADCs, this method led to stable conjugates with potent anti-tumour efficacy in xenograft mouse models of cancer.^88^ A Knoevenagel condensation strategy was also used by the same researchers to modify aldehyde-tagged mAbs. This method uses a pyrazolone-stabilized carbanion for nucleophilic attack, followed by dehydration to produce an enone, which is then trapped by a thiol nucleophile (Fig. 7c).^79^ The resulting plasma-stable ADCs showed potent *in vivo* activity in models of cancer in mice.^79^

The genetic encoding of non-canonical amino acids in mAbs allows for the use of novel chemistries and the production of homogenous ADCs. Thus, the introduction of unique functionalities, especially aldehydes and ketones, and the development of chemistries targeting these handles, offer great promise for building chemically-defined ADCs with improved safety and efficacy profiles.

## Drug conjugation through glycoengineering

The conserved, glycosylated Asn 297 residue in the CH2 domain of an IgG mAb provides yet another alternative for the linking of cytotoxic payloads to antibodies. Indeed, a number of strategies that use glycoengineering, followed by chemical ligation, have been explored for the attachment of drugs to mAbs.^12^

One of the first methods involved periodate oxidation of the glycan motif and then reaction with hydrazide toxic moieties to form a hydrazone-linked conjugate.^89^–^91^ More recently, Senter and co-workers investigated the metabolic incorporation of modified fucose (Fuc) analogues at the terminus of the *N*-glycan present in IgGs and the use of these analogues for conjugation.^92^ They showed that fucosyltransferase VIII efficiently incorporated a variety of non-natural sugars into the antibody carbohydrate, including the thio-sugar 6-thiofucose. Maleimide chemistry was then used to link the antibody with MMAE *via* 6-thiofucose. This strategy resulted in an ADC with a DAR of ∼1.3 and with enhanced plasma stability when compared to an ADC prepared using Cys/maleimide conjugation chemistry.^92^

**Fig. 8:**
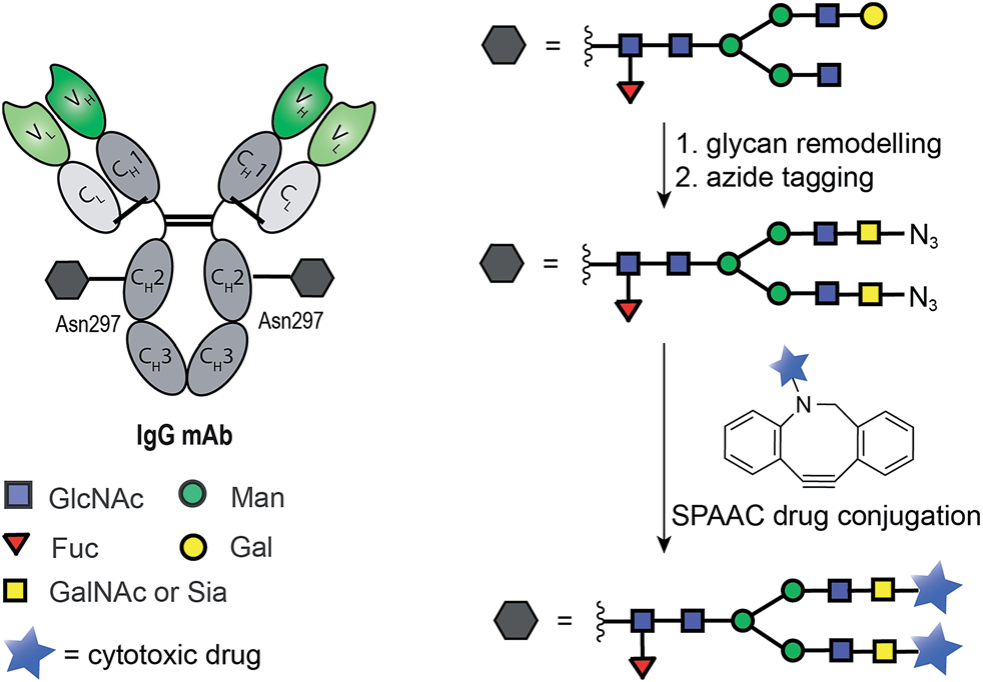
General chemoenzymatic strategy for drug conjugation to the conserved glycan of mAbs for the construction of homogenous ADCs. The strategy consists of glycan remodelling that allows the incorporation of non-natural azide-tagged carbohydrate motif (GalNAc^94^ or Sia^93^) followed by SPAAC ligation with a suitable payload.

The ability of certain glycosyltransferases to tolerate modifications of their sugar nucleotide substrates has also been explored as a means of introducing reactive functionalities for antibody drug-conjugation. One strategy requires glycan trimming and modification with an azide-tagged carbohydrate, followed by SPAAC ligation of a cytotoxic derivative (Fig. 8). Boons and co-workers used a cytidine monophosphate sialic acid displaying an azide at C-9 to tag the glycan motif of a CD22 mAb. A SPAAC reaction at this azide was then used to install a doxorubicin derivative, and the product ADC was shown to selectively target and kill lymphoma cells *in vitro* (Fig. 8).^93^ Delft and co-workers incorporated an azido-modified GalNAc into trastuzumab and used a SPAAC reaction to conjugate the cytotoxic maytansine instead. Notably, in carrying out this conjugation, the authors found that bicyclononyne (BCN) is more efficient than dibenzoannulated cyclooctyne (DBCO) reagents when conjugation to the azido-modified GalNAc derivative is the goal.^94^

Azide motifs can also be introduced to the glycan moieties of antibodies using a chemoenzymatic approach. Lai-Xi Wang and co-workers have developed a method of site-selective Fc glycoengineering for the transfer of predefined *N*-glycans from corresponding glycan oxazolines to the Fc-deglycosylated intact IgGs, an approach used to display azides.^95^ Finally, glycan remodelling of terminal sialic acids has also been combined with periodate oxidation to allow for conjugation with aminooxy functionalized cytotoxic agents.^96^

Modification of IgG glycan motifs, followed by chemical drug conjugation, has proven to be a useful strategy for the creation of homogenous and stable ADCs. However, the impact of remodelling the glycan motif on the immunogenicity of conjugates is still not clear.^97^ An analogue of the naturally occurring sialic acid, *N*-glycolylneuraminic acid, was shown to act as an antigen *in vivo*.^98^ Moreover, far fewer drugs can be linked to a mAb through glycan remodelling than through direct amino acid conjugation. These limitations mean that few ADCs produced *via* glycoengineering conjugation strategies have reached clinical studies.

## Conclusions

Since the first generation of ADCs, advances in antibody engineering and a growing variety of linkers and strategies for drug-release have enabled the development of ADCs with improved pharmacokinetic profiles. Of particular importance has been the use of site-selective chemical conjugation methodology to address a critical cause of limited efficacy and safety: heterogeneity. The development of new methodologies and the optimization of old has inspired a generation of homogenous ADCs. These ADCs, such as one developed by Senter and co-workers, have proven to be therapeutically effective even with a relatively high DAR of 8 – upending the idea that a DAR of 2 to 4 is optimal and suggesting that selective, controlled conjugation of more drug molecules increases therapeutic potential.^61^ Thus, the introduction of homogeneity *via* selective chemical conjugation is crucial in refining the pharmacokinetics, toxicity, antigen affinity/selectivity, and drug release properties of ADCs.

Chemical site-selective conjugation methodologies not only provide a path to ADCs with a higher DAR, they could also enable the construction of multifunctionalized adducts – that is, a single antibody bearing two cytotoxic molecules. Recently, Chudasama, Caddick and co-workers inserted pyridazinediones bearing orthogonal ‘clickable’ handles into the native disulfide bonds of trastuzumab.^68^ Two orthogonal transformations allowed for the introduction of both a drug (Dox-N_3_) and a fluorophore (sulfo-Cy5-N_3_). This work established the principles for the synthesis of dual-modified ADCs. Now, the creation and *in vivo* evaluation of ADCs bearing a double payload are required for development of next-generation ADCs.

The application of chemical site-selective conjugation strategies to the construction of ADCs has improved not only the stability and homogeneity but also the flexibility of ADCs. Thus, ADCs remain at the vanguard of targeted therapeutics, offering the promise of a novel, effective treatment for cancer.
